# Self-Administered Ethanol Enema Causing Accidental Death

**DOI:** 10.1155/2014/191237

**Published:** 2014-11-11

**Authors:** Thomas Peterson, Landen Rentmeester, Bryan S. Judge, Stephen D. Cohle, Jeffrey S. Jones

**Affiliations:** ^1^College of Human Medicine, Michigan State University, Grand Rapids, MI 49503, USA; ^2^GRMEP/Michigan State University Program in Emergency Medicine, Grand Rapids, MI 40503, USA; ^3^Department of Pathology, Spectrum Health Hospitals, Grand Rapids, MI 49506, USA

## Abstract

Excessive ethanol consumption is a leading preventable cause of death in the United States. Much of the harm from ethanol comes from those who engage in excessive or hazardous drinking. Rectal absorption of ethanol bypasses the first pass metabolic effect, allowing for a higher concentration of blood ethanol to occur for a given volume of solution and, consequently, greater potential for central nervous system depression. However, accidental death is extremely rare with rectal administration. This case report describes an individual with klismaphilia whose death resulted from acute ethanol intoxication by rectal absorption of a wine enema.

## 1. Introduction

Nearly one-third of US adults consume enough alcoholic beverages to cause or place them at risk of adverse consequences. According to the Centers for Disease Control and Prevention, nearly 5% of the US population drinks heavily, and 15% engages in risky, binge drinking patterns that could lead to ethanol problems [1]. Most binge drinkers are not ethanol dependent, and they often engage in this health risk behavior without realizing the problems associated with drinking excessively [1]. Reckless behaviors such as combining ethanol with prescription drugs or ingesting ethanol through the rectum increase the effects of ethanol and can pose even greater risks than binge drinking. It is unclear when ethanol enemas first appeared or how frequently they are being used. However, this appears to be a dangerous new trend, particularly among college students [2]. A tube is inserted in the rectum and ethanol is poured directly into the colon enhancing the amount and speed of the ethanol entering the bloodstream as it bypasses the initial filtering by the liver. Some participants may be under the impression that this practice prevents vomiting and hangovers; others are unable to ingest ethanol orally because of painful gastrointestinal ailments [2].

Another group using ethanol enemas practice klismaphilia, receiving sexual arousal from introducing liquids into the rectum and colon via the anus. It is a paraphilia that often involves the use of enemas [3]. The term klismaphilia was coined in 1973 by Dr. Denko, an early investigator in this field, to describe the activities of some of her patients. Typically, it is warm water that is used to clean the lower rectum although other substances have been reported including coffee, yogurt, air, whisky, wine, beer, cocaine, and even epoxy resin [4]. Hemandas and colleagues note that the incidence of complications due to enema overuse and abuse may increase due to increasing popularity of various erotic practices and sexual exploration [4].

## 2. Case Presentation

A 52-year-old male was found unresponsive in his home by a coworker performing a welfare check due to unexplained work absence. He was last seen alive 66 hours prior to discovery. His medical history consisted only of hypertension, treated with metoprolol and valsartan. Emergency medical services were summoned, and on arrival the presence of a middle aged white male, lying on his left side in bed, was noted. The patient was in fixed rigor and livor mortis, was asystolic, and was pronounced dead. An enema bag was hanging from a coat rack next to the bed and contained a yellow colored fluid. A tube was connected to the enema bag and the other end inserted into the patient's rectum. Several empty boxes of white wine were also discovered during the scene investigation. The patient was wearing a condom and women's underwear, and numerous pornographic materials were found throughout the room. The patient was transported to a nearby hospital and an autopsy was performed that day.

Pertinent autopsy findings included no external injury or indicia of IV drug abuse. Internal examination revealed pulmonary congestion and edema, fatty change of the liver, and 500 mL of yellow fluid in the colon, of the same color and consistency as that in the enema bag. The toxicology screen revealed a blood ethanol concentration of 350 mg/dL and a vitreous ethanol concentration of 410 mg/dL. Analysis of the liquid in the enema bag revealed the presence of alcohol and the liquid was concluded to be white wine. The cause of death was acute ethanol intoxication by ethanol enema.

## 3. Discussion 

Rectal administration of ethanol has been described in eight previous case reports [5–13]. In these reports, patients either intentionally or accidentally introduced varied ethanol solutions ranging from 35 to 95% ethanol content. Symptoms usually develop within 24 hours of introduction and include anal pain, tenesmus, and colonic bleeding [11]. Endoscopic findings are often nonspecific and range from edema with loss of vascular markings to friable, hemorrhagic, or necrotic mucosa with pseudomembranes and ulcers. Histologic features are also nonspecific, and the diagnosis may not be entertained if the clinician and pathologist are not cognizant of the agent and circumstances leading to the colonic biopsy. Despite the severity of the injury to the colonic lumen and the clinical features, almost all reported patients recovered completely in 7 to 10 days with conservative management. The only reported fatality was similar to our case: acute ethanol intoxication by rectal absorption of a wine enema [13]. The postmortem analysis in our patient noted significant pulmonary congestion and edema. This finding was also present in the other case report of lethal wine enema [13] and likely represents noncardiogenic pulmonary edema from ethanol-induced respiratory depression and carbon dioxide narcosis.

Of the intentional cases, two were discovered to be in association with sexual fetishes [7, 11], the third case was the result of rectal administration of a solution with an extremely high ethanol content for the purpose of hemorrhoid relief [9], and the fourth case used ethanol in an effort to improve bowel cleansing [12]. Our case is remarkable not only in the fact that the rectal absorption of ethanol proved to be lethal to the patient, but also because prior case reports of ethanol-containing enemas described mucosal damage on endoscopy. The intact colonic mucosa in our patient suggests that the ethanol content of wine is below the threshold for inducing gastrointestinal mucosal irritation or that the patient died before chemical colitis could develop. Acute colitis due to intrarectal administration of ethanol is uncommonly reported and likely underrecognized [10].

The importance of this case also lies in reminding the clinician of the significant consequences inherent to ethanol intoxication and how the route of ethanol absorption plays a role in its clinical effects. Metabolism of ethanol begins as soon as it reaches the gastric mucosa. Rectal absorption of ethanol bypasses the first pass metabolic effect, allowing for a higher systemic concentration of blood ethanol to occur for a given volume of solution prior to hepatic degradation and, consequently, greater potential for central nervous system depression [2, 5]. In fact, rectal absorption of drugs is the basis for the administration of medications such as diazepam, in individuals who need emergency management of seizures when oral or intravenous therapy is not an option [13].

A significant proportion of ethanol related morbidity is associated with excessive episodic or “binge” drinking, especially in intolerant users. Ninety percent of ethanol consumed by underage drinkers is consumed while binge drinking, according to the Centers for Disease Control [1]. Equally alarming are the new forms of ethanol exposure being devised, such as ethanol-soaked tampons, vodka eyeballing, funneling, and ethanol inhalation. These alternative routes of alcohol use are being popularized in social and entertainment media and appear to be a dangerous new trend among college students [2].

## 4. Conclusion

Despite the plethora of alcoholic beverages available to the public, one common factor is the fact that they are all manufactured for oral consumption. Practitioners should be mindful that ethanol intoxication may occur through additional routes and that the degree of intoxication may be less predictable. We report what we believe to be the second case of lethal ethanol intoxication resulting from the rectal administration of a wine enema. Fluids with lower ethanol content may be less likely to induce colonic inflammation; however, rapid absorption of ethanol through the colonic mucosa can lead to significant CNS depression and possibly death.

## References

[1] Centers for Disease Control and Prevention (2014). *Alcohol and Public Health*.

[2] Wilson J. (2012). Experts: alcohol enemas “extremely dangerous”. *CNN Health*.

[3] Denko J. D. (1973). Klismaphilia: enema as a sexual preference. Report of two cases. *The American Journal of Psychotherapy*.

[4] Hemandas A. K., Muller G. W., Ahmed I. (2005). Rectal impaction with epoxy resin: a case report. *Journal of Gastrointestinal Surgery*.

[5] Herrerias J. M., Muniain M. A., Sanchez S., Garrido M. (1983). Alcohol-induced colitis. *Endoscopy*.

[6] Rodriguez-Tellez M., Herrerias J. M., Argülles F., Gonzalez-Mariscal M. J., Pellicer F. J. (2001). Accidental intrarectal administration of alcohol enema induces proctocolitis and fecal incontinence. *Endoscopy*.

[7] Kawamura O., Sawada T., Hara T., Kodama T., Sanada H., Simazaki S., Mugikura M., Nishida Y., Ohhara H., Matsushita K. (2002). Chemical proctocolitis due to alcohol (Shochu) enema. *Gastroenterological Endoscopy*.

[8] Bhalotra R. (1988). Alcohol-induced proctitis in a human. *Journal of Clinical Gastroenterology*.

[9] Triantafillidis J. K., Vekini J., Nicolakis D., Emmanouilides A. (1994). Ethanol-induced proctitis: another kind of chemical proctitis. *The American Journal of Gastroenterology*.

[10] Michopoulos S., Bouzakis H., Sotiropoulou M., Papaspyrou I., Tsibouris P., Kralios N. (2000). Colitis due to accidental alcohol enema: clinicopathological presentation and outcome. *Digestive Diseases and Sciences*.

[11] Mian S. R., McFadden K. L., Valenstein P. N., Shehab T. M. (2005). Self-administered alcohol (vodka) enema causing severe colitis: case report and review. *Gastrointestinal Endoscopy*.

[12] Randolph M., Longacre T. A., Gerson L. B. (2005). Acute colitis secondary to self-administered alcohol enemas: a mimic of ischemic colitis. *Journal of Clinical Gastroenterology*.

[13] Wilson C. I., Ignacio S. S., Wilson G. A. (2005). An unusual form of fatal ethanol intoxication. *Journal of Forensic Sciences*.

